# Association between Climatic Variables and Malaria Incidence: A Study in Kokrajhar District of Assam, India: Climatic Variables and Malaria Incidence in Kokrajhar District

**DOI:** 10.5539/gjhs.v5n1p90

**Published:** 2012-11-11

**Authors:** Dilip C. Nath, Dimacha Dwibrang Mwchahary

**Affiliations:** 1 Department of Statistics, Gauhati University, Assam, India

**Keywords:** monthly malaria incidence rate, climatic variables, forest area, non-forest area, modeling

## Abstract

A favorable climatic condition for transmission of malaria prevails in Kokrajhar district throughout the year. A sizeable part of the district is covered by forest due to which dissimilar dynamics of malaria transmission emerge in forest and non-forest areas. Observed malaria incidence rates of forest area, non-forest area and the whole district over the period 2001-2010 were considered for analyzing temporal correlation between malaria incidence and climatic variables. Associations between the two were examined by Pearson correlation analysis. Cross-correlation tests were performed between pre-whitened series of climatic variable and malaria series. Linear regressions were used to obtain linear relationships between climatic factors and malaria incidence, while weighted least squares regression was used to construct models for explaining and estimating malaria incidence rates. Annual concentration of malaria incidence was analyzed by Markham technique by obtaining seasonal index. Forest area and non-forest area have distinguishable malaria seasons. Relative humidity was positively correlated with z malaria incidence, while temperature series were negatively correlated with non-forest malaria incidence.

There was higher seasonality of concentration of malaria in the forest area than non-forest area. Significant correlation between annual changes in malaria cases in forest area and temperature was observed (coeff=0.689, p=0.040).

Separate reliable models constructed for forecasting malaria incidence rates based on the combined influence of climatic variables on malaria incidence in different areas of the district were able to explain substantial percentage of observed variability in the incidence rates (R^2^_adj_=45.4%, 50.6%, 47.2%; p< .001 for all). There is an intricate association between climatic variables and malaria incidence of the district. Climatic variables influence malaria incidence in forest area and non-forest area in different ways. Rainfall plays a primary role in characterizing malaria incidences in the district. Malaria parasites in the district had adapted to a relative humidity condition higher than the normal range for transmission in India. Instead of individual influence of the climatic variables, their combined influence was utilizable for construction of models.

## 1. Introduction

Association between malaria incidence and climatic behavior has been established through various malaria studies conducted for different parts of the world. Several studies have revealed that there may be influence of climatic variables on malaria transmission in either way. During 1988-1999, in Zimbabwe, inter-annual variations in average temperature, rainfall and vapour pressure were found to have strong positive association with malaria incidence while maximum and minimum temperature were acting in opposite way (Musawenkoi et al., 2006). Climate variability was found to play an important role in initiating malaria epidemics in the East African highlands (Zhou et al., 2004). Jones et al. (2007) found that high malaria incidence was associated with increased rainfall and high maximum temperature in Tanzania (Anne et al., 2006). Though malaria had been found to be associated with some socio-economic factors (Niringiye & Douglason, 2010; Sheila et al., 2009), climatic factors are primary ones as these can play direct role in the development of malaria mosquitoes and the transmissions of the parasite (Huang et al., 2001). Significant correlations between malaria incidence rate and climatic factors were obtained for four Governorates of Yemen also (Al-Mansoob et al., 2005). Results of some studies suggested that instead of instantaneous effect of climatic factors on malaria incidence, there might be lagged effect (Alemu et al., 2011). Malaria incidence was found to be lagging zero to three months behind rainfall in Sri Lanka (Briët et al., 2008). A 12 year data analysis on malaria incidence and climatic factors in Shuchen County in China suggested that climatic variables should be considered as possible predictors of malaria (Peng et al., 2003). Considering their significant roles on malaria transmission, climatic factors are now incorporated as explanatory variables in most of the new systems developed for forecasting malaria incidence. One such worth mentioning pioneer model was that of Loevinsohn (1994), in which expected malaria rate was expressed in terms of climatic variables observed in preceding months. It was found that the models incorporating climatic variables as predictor variables could provide a little more improved forecasting over those without them (Briët et al., 2008).

In spite of broad studies on the association between climatic variables and malaria incidence in different parts of the world, such literatures from India are very limited (Devi & Jauhari, 2006). A study for Dehradun of Uttaranchal demonstrated high positive association between climatic variables and malaria incidence (ibid.). Another study for Sonitpur district of Assam revealed that densities of malaria vectors were influenced by rainfall pattern (Baruaha et al., 2007).

## 2. Study Area

Kokrajhar, located in the state of Assam of North-east India, is a malaria prone district, which has been identified by the National Vector Born Disease Control Programme (NVBDCP) of the country as one of the eight malaria endemic districts in the state. The district contributes more than 6 % to the state malaria cases. A major part of the district lying in the north is covered by forests where the inhabitants are socio-economically backward tribal people. The villages inside the forests are scattered, thinly populated and backward in communication. These villages are difficult to approach and remain inaccessible by road during rainy season. About 33% of the total population of the district belongs to backward Scheduled Tribe category and 36.09% of the people live under poverty line (Kokrajhar DRDA, 2010).

The district has been endemic disease ridden area since old times. In the twenties of the 20th Century, an endemic disease called Kala-azar used to sweep through the district resulting in a decimation of people (Jacob, 1939). This disease no longer exists; other vector borne diseases have become very rare in the district now-a-days. However, malaria remains to be endemic in the district and creates havoc among the masses. Malaria incidences keep on fluctuating in the district without any clear trend. Plasmodium falciparum (PF) and Plasmodium vivax (PV) are the only active malaria parasites in the district, and PF is dominant one. Malaria is more prevalent in forest area than non-forest area of the district (Nath & Mwchahary, 2012).

**Figure 1:**
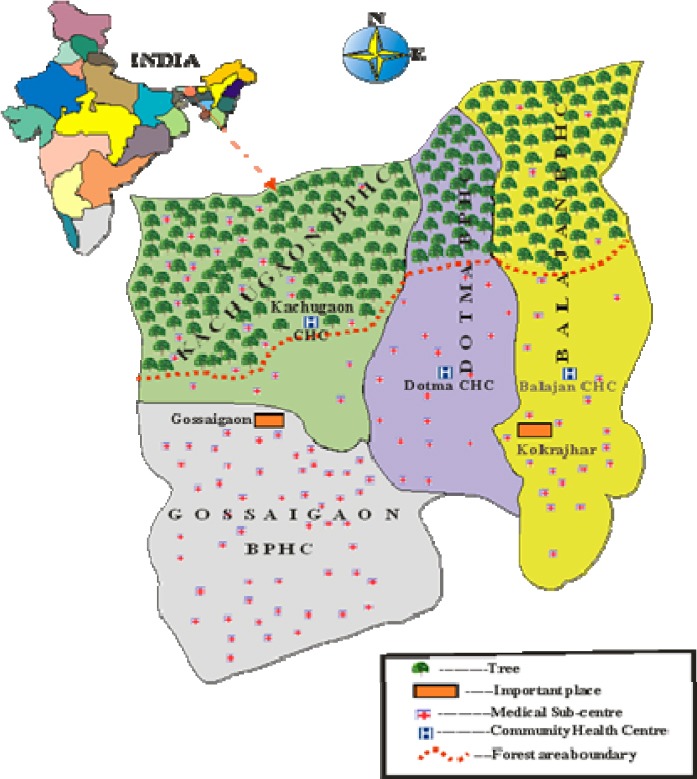
Medical blocks of Kokrajhar district

The district consists of four medical blocks, namely Kachugaon, Gossaigaon, Dotma and Balajan; these are called Block Public Health Centres (BPHC). Almost the whole area of Kachugaon Block Primary Health Centre lies within forest area, while little parts of Dotma and Balajan BPHCs are also included within forest area (Figure 1). The term “Forest Area” generally refers to all the geographic areas recorded as forest in government records and comprises Reserved Forests and Protected Forests, which have been constituted under the provisions of Indian Forest Act, 1927 (SFR, 2009).

During the period 2001-2010, among the four BPHCs of the district, Kachugaon recorded the highest mean (13.94%) of slide positivity rate (SPR, percentage of malaria positive cases over total blood slides examined), followed by Gossaigaon (5.49%), Balajan (5.33%) and Dotma (3.59%). The four BPHCs followed the same sequential order of SPR in annual parasite incidence (API, malaria positive cases per thousand populations) also, their mean APIs being 12.64, 7.43, 5.42 and 3.53.

No entomological survey has been conducted in the district till now, and as such the identity of the different vector species present in the study site areas could not be ascertained. However, from the field study of the vector borne disease sites, it may be remarked that An. Dirus and An. Minimus malaria vectors and Cx. vishnui Japanese encephalitis vectors are active in the district (S. Kakoti, District Malaria Officer, personal communication).

## 3. Materials and Methods

### 3.1 Materials

#### 3.1.1 Data

Monthly malaria incidence and climatic variable records over the period 2001-2010 were considered for analysis of their temporal correlation. Records of monthly malaria incidence were collected from the office of the Kokrajhar District National Vector Borne Disease Control Programme. Monthly rainfall and rainy days records were collected from two sources: Kokrajhar District Agriculture Office, Kokrajhar and Regional Agricultural Research Station, Gossaigaon of the same district. Temperature and relative humidity data were collected from Indian Meteorological Department, Regional Meteorological Centre, Guwahati, recorded at Rupsi Station, which is situated within the study district.

#### 3.1.2 Climatic Seasons

The state of Assam possesses a tropical monsoon rainforest climate and so is Kokrajhar district. Based on climatic condition of the district, the whole year may be divided into two seasons: the summer, and the winter. Summer starts from the month of April and ends in September. It covers the monsoon season of the district that starts in June and ends in September. Winter starts in October and continues up to March.

#### 3.1.3 Annual Incidence of Malaria in Kokrajhar District

Annual malaria incidence rate ranged from 5.18 per 1,000 population in 2008 to 10.2 per 1,000 population in 2002 over the period 2001-2010 (Figure 2) in the district. High endemic occurred in the years 2002 and 2006. Malaria incidence in 2010 had invalidated the idea of malaria epidemic after a period of four years, as there was no epidemic that year. There was no clear trend of malaria incidence, and though recession had been observed in the later part of the period, the consistency of reduction is incomprehensible.

**Figure 2a:**
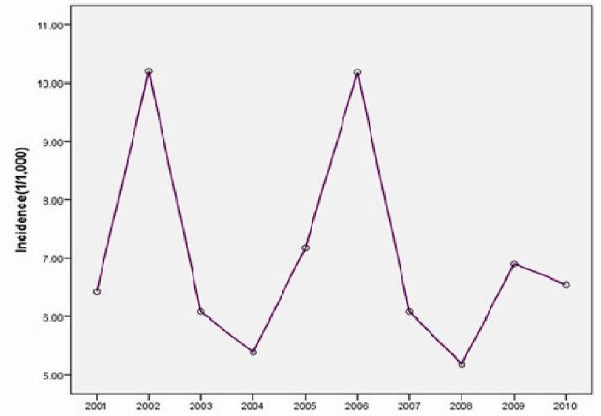
Annual incidences of malaria of the district

**Figure 2b:**
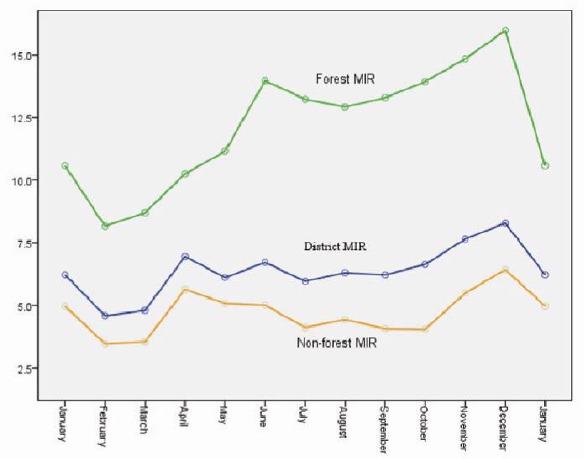
Monthly mean malaria incidences in the district and forest & non-forest areas

#### 3.1.4 Pattern of Monthly Malaria Incidence

Over the period 2001-2010, malaria was found prevailing throughout the year in the district. The lowest and the highest mean incidence rates of malaria of the forest area, non-forest area, and the district occurred in the same months that are in February and December respectively. Malaria incidences in forest area and non-forest area were found following different patterns.

Two malaria seasons may be observed in the forest area: the first season from February to July and the second one from August to January. In the first season, malaria incidence started to increase gradually from the yearly lowest and attained seasonal highest rate in the month of June, and then it started to decline. In the second season, malaria incidence started to increase from the seasonal lowest in August, kept on increasing, then attained the seasonal as well as yearly highest incidence rate in the month of December, and then took to decline again.

On the other hand, based on the pattern of malaria incidence in the non-forest area, the whole year may be divided into three malaria seasons- 1) pre-monsoon season, from February to May, 2) monsoon season, from June to September, and 3) post-monsoon season from October to January. There were two notable peaks of MIR in the year, and they occurred in pre-monsoon and post monsoon seasons, the post-monsoon peak being more pronounced than the other. During the pre-monsoon season, malaria started to rise from its lowest rate in February, attained its seasonal highest during April with a gradual increase, and then took to decline. During the monsoon season, malaria incidence fluctuated within intermediate range. Finally, during the post-monsoon season, the malaria incidence started to rise stiffly from intermediate rate to attain the yearly highest rate during the month of December, and then it came down again all of a sudden (Figure 2). Malaria of the whole district was found to follow the same pattern as that of non-forest area.

#### 3.1.5 Climatic Variables and Their Patterns

3.1.5.1 Rainfall

Kokrajhar district received heavy annual rainfall during the last ten years from 2001 to 2010, but some months went without any rain in winter sometimes. Over the period, the lowest rainfall occurred during the month of December while the month of July received the highest rainfall. The rainfall seemed to increase gradually from the month of January, occurred highest in the month of July and then declined gradually to its lowest in the month of December. The months of November, December and January were dull months of rainfall, each of them having only one day rainfall in average.

3.1.5.2 Temperature

January was the coldest month of the year in the district while August was the hottest month. Temperature took to rise gradually after January, attained its highest in the month of August, and then kept on falling down up to December. Thus, rainfall led temperature by a month in the district.

3.1.5.3 Relative Humidity

Relative humidity of the district initially declined from January to attain the lowest value in March and then kept on increasing. The highest humidity was attained in the month of July. The highest rainfall as well as the highest humidity occurred in the same month, the July.

Figures 3a, 3b, 3c show the monthly patterns of climatic variables in the district over the study period.

**Figure 3a:**
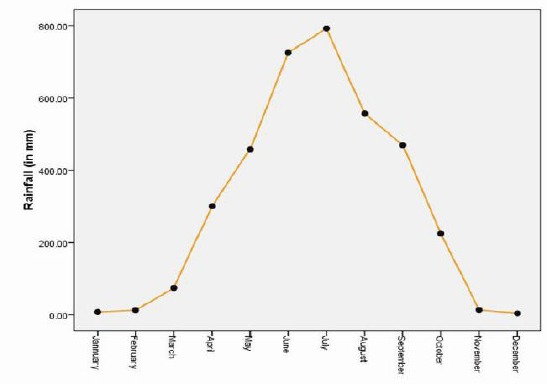
Monthly mean rainfall

**Figure 3b:**
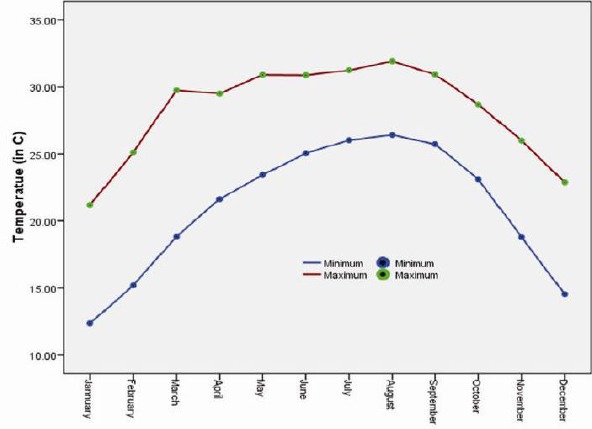
Maximum and minimum temperatures

**Figure 3c:**
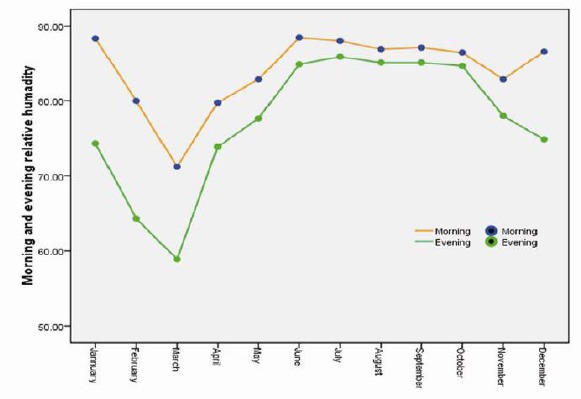
Morning and evening humidity

## 3.2 Methods

Since almost the whole part of Kachugaon BPHC lies within the forest area, its malaria incidence was considered as the representative of malaria incidences in forest area. On the other hand, the mean of the three other BPHCs was considered as malaria incidence in non-forest area. From the malaria data, malaria incidences for forest and non-forest areas were separately extracted. Monthly malaria incidence rate (MIR) for the whole district, forest area and non-forest area was calculated by the formula-


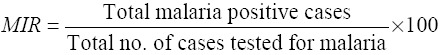


and then three respective MIR time series were obtained. These malaria incidence series were considered for analyzing their statistical relationships with climatic variables, namely temperature, rainfall, rainy days and relative humidity. Mean of the rainfall records from two stations were considered as rainfall of the district. For the analysis, statistical softwares R (2.15) and SPSS (16) were used. In order to examine the association between monthly malaria incidence rates and climatic variables, Pearson correlation analysis was conducted. The time lag(s) of climatic factors preceding malaria at which the series showed the strongest correlation were obtained by cross-correlation analysis of monthly malaria incidence series and monthly climatic data time series. Cross-correlation tests were performed between residuals of pre-whitened series of climatic variables and residuals of malaria series. For this purpose, seasonality and auto-correlation of climatic data series were removed by employing multiplicative seasonal auto-regressive integrated moving average (SARIMA) models to the climatic variable series first and then the same model was employed to the malaria series also; and residuals were collected from both series.

Cross-correlation analysis was conducted with the seasonally adjusted series also. Seasonally adjusted series of all variables, except rainfall and rainy days, were obtained by multiplicative model, while that of rainfall and rainy days were obtained by additive model as these contained zero values for some observations.

An inter-annual analysis was carried out to find underlying relationship between malaria data series and meteorological data series. Analysis was conducted with differenced annual series of malaria of three areas, and three climatic factors namely mean temperature, rainfall and mean relative humidity were considered. For this purpose, monthly values of climatic variables were accumulated and then averages were termed as mean annual values; on the other hand, mean malaria incidence rates were calculated from actual yearly cases.

Another inter-annual analysis was made applying Markham technique (Markham, 1970; Musawenkoi et al., 2006) for determining the months with most malaria seasonality. Seasonality indices of malaria and climatic variables were obtained for all the years of study period.

Linear relationships between climatic variables and malaria series were obtained by linear regression. Weighted least squares regression was used to construct models with climatic variables for estimating malaria incidences in the three areas.

## 4. Modeling Assumptions for Influence of Climatic Variables on Malaria

The influence of climatic factors on the malaria incidence rate of an area, according to Gomez-Elipe et al. (2007), can be explained by the relation-


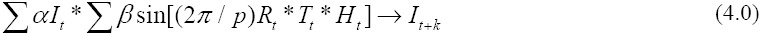


where I_t_ is the incidence rate at time *t*; R_t_, T_t_, H_t_ are climatic variables at time *t*, which are rainfall, mean temperature and mean relative humidity for the current study, *p* is the seasonal period of oscillation of these climatic variables, and I_t+k_ is the incidence rate at time *t+k*, associated with some suitable arithmetic operation (*) and parameters α and β.

For modeling malaria incidence rates of the three areas, viz. forest area, non-forest area and the whole district, data for the period 2001-2009 was considered and rest data for the year 2010 was used for validation of the models.

## 5. Results

### 5.1 Association between Climatic Variables and Monthly Malaria Incidence

Pearson’s correlation analysis demonstrated that climatic variables of the district were highly correlated with each other at 1% significance level. All correlations were positive and the highest correlation was found to exist between minimum temperature and rainfall (coefficient= .712).

None of the climatic variable was found to bear a significant correlation with monthly malaria incidence rate of the whole district (DMIR) at 5% significance level, only the mean and morning relative humidity bore a significant correlation at 10% significance level. All the temperature and rainfall components were negatively correlated with DMIR, while correlations exhibited by relative humidity components were positive.

Temperature components exhibited mixed up correlations with monthly malaria incidence rate of the forest area (FMIR); minimum and mean temperatures were positively correlated, while maximum temperature was negatively correlated. Rainfall was also negatively correlated with FMIR. However, all the relative humidity components were significantly correlated at 5% significance level, and correlations were positive.

The nature of correlations between malaria incidence of non-forest area (NFMIR) and climatic variables was almost same as that between the DMIR and climatic variables. However, maximum and mean temperatures were significantly correlated with NFMIR at 5% significance level with negative correlations.

### 5.2 Season-Wise Correlations between MIR and Climatic Variables

When the association between FMIR and climatic variables was analyzed season wise, all the climatic variables, except maximum temperature, exhibited significant positive correlations with FMIR in the first malaria season. However, in the second season, the association was completely reversed to obtain negative correlations of FMIR with all climatic variables, except minimum temperature, and none of the correlation was significant (Table 1).

**Table 1:** Season wise correlations between MIR and climatic variables

MIR	CV	Season-I		Season-II		Season-III	
		Coeff.	Sig.	Coeff.	Sig.	Coeff.	Sig.
FMIR	T_min_	0.387**	0.002	0.004	0.976	-	-
	T_max_	0.210	0.107	-0.011	0.935	-	-
	T_mean_	0.332**	0.010	-0.004	0.978	-	-
	RF	0.452**	0.000	-0.068	0.604	-	-
	RD	0.429**	0.001	-0.013	0.923	-	-
	RH_m_	0.339**	0.008	-0.151	0.250	-	-
	RH_e_	0.375**	0.003	-0.095	0.471	-	-
	RH_mean_	0.368**	0.004	-0.139	0.291	-	-

NFMIR	T_min_	0.377*	0.016	-.332*	0.036	-0.325*	0.041
	T_max_	0.162	0.316	-0.150	0.356	-0.349*	0.027
	T_mean_	0.296	0.064	-0.250	0.119	-0.338*	0.033
	RF	0.363*	0.021	0.095	0.562	-0.264	0.100
	RD	0.404**	0.010	0.140	0.390	-0.250	0.120
	RH_m_	0.223	0.166	-0.172	0.288	-0.198	0.221
	RH_e_	0.369*	0.019	-0.163	0.316	-0.433**	0.005
	RH_mean_	0.324*	0.041	-0.187	0 .248	-0.448**	0.004

DMIR	T_min_	0.378*	0.016	-0.196	0.225	-0.054	0.741
	T_max_	0.171	0.292	-0.129	0.427	-0.096	0.558
	T_mean_	0.299	0.061	-0.170	0.294	-0.073	0.656
	RF	0.285	0.074	0.007	0.965	0.045	0.783
	RD	0.396*	0.011	0.080	0.623	-0.005	0.973
	RH_m_	0.233	0.148	-0.179	0.268	-0.118	0.468
	RH_e_	0.348*	0.028	-0.188	0.246	-0.165	0.309
	RH_mean_	0.315*	0.048	-0.205	0.205	-0.190	0.240

NFMIR was also found to bear positive correlations with all the climatic variables in the pre-monsoon season, and correlations with minimum temperature, rainfall, rainy days and evening and mean relative humidity were significant. In the next season, which was the monsoon, only minimum temperature bore a significant correlation; rainfall and rainy days continued to bear positive correlations, but correlations of other variables were changed from positive to negative. In the post-monsoon malaria season, all correlations became negative; and correlations of all temperature components and evening & mean relative humidity with NFMIR were significant.

The nature of correlations between DMIR and climatic variables was almost same as the correlations between climatic variables and NFMIR. Notable differences were that correlation of DMIR with rainfall remained positive in all the three malaria seasons, but all of them were insignificant, and minimum temperature ceased to bear significant correlations in the last two seasons.

### 5.3 Cross-Correlations between MIR and Climatic Variables

All the malaria and climatic variables exhibited autocorrelation. Hence, original series were employed suitable seasonal arima models to obtain white noise residuals, and then the DMIR, FMIR and NFMIR series were filtered with the same arima model. Finally, cross-correlation tests were performed between residual series of the climatic variables and the respective residual series of the MIR. With an aim to study the influence of climatic variables on malaria incidence, stress was given only on the significant correlations of climatic variables with malaria incidence lagging up to ten months.

Cross-correlation analysis of temperature components with MIRs showed that minimum temperature was not significantly correlated with both DMIR and FMIR, but it bore a significant correlation with NFMIR at lag (-1). Maximum temperature was not found to exhibit significant correlations with both DMIR and FMIR up to lag (-7); however, fair correlations with both these MIRs were observed at lag (-9), while NFMIR did not bear any significant correlation with maximum temperatures preceding it. Mean temperature exhibited a fair correlation with NFMIR lagging one month, but no significant correlation was found to occur between mean temperature and MIRs of the whole district and the forest area.

Rainfall and rainy days did not exhibit any significant correlation with all the components of MIR at any lag. Only the morning relative humidity was found to bear significant correlations with FMIR and NFMIR at the lags of (-9) and (-10) respectively; no other significant correlations between components of relative humidity and MIR was found to exist.

Almost the same outcomes were obtained when cross-correlations were performed by seasonally adjusted series; the only additional outcome was that of a significant correlation of non-forest malaria incidence with minimum temperature preceding one month.

From the cross-correlation analysis, it could be concluded that temperature had delayed impact on malaria incidences of the whole district and the forest area, while it had an immediate consequent impact on malaria incidence in non-forest area. Relative humidity also rendered delayed impact on malaria incidences. On the other hand, the influence of rainfall and rainy days on malaria incidences in all areas was not explicit.

### 5.4 Inter-Annual Analysis

Analysis of differenced series of mean annual malaria cases of the whole district, forest area and non-forest area, annual averages of rainfall, mean temperature and mean relative humidity, showed that annual changes in malaria cases in forest area and temperature were significantly correlated (coeff=0.689, p=0.040); there was no other significant correlation. There was also no significant correlation between annual changes of climatic variables and changes in malaria cases one to three years behind climatic variables. Analysis of relative changes of the above variables obtained by the technique (X_t_ −X_t-1_)/X_t-1_ (“http://www.duke.edu/~rnau/simpreg.Htm #model5”), revealed that percentage change in temperature was significantly correlated with percentage changes of malaria cases in both the forest area and the whole district (correlations are (0.780; 0.013) and (0.682; 0.043) respectively).

**Figure 4:**
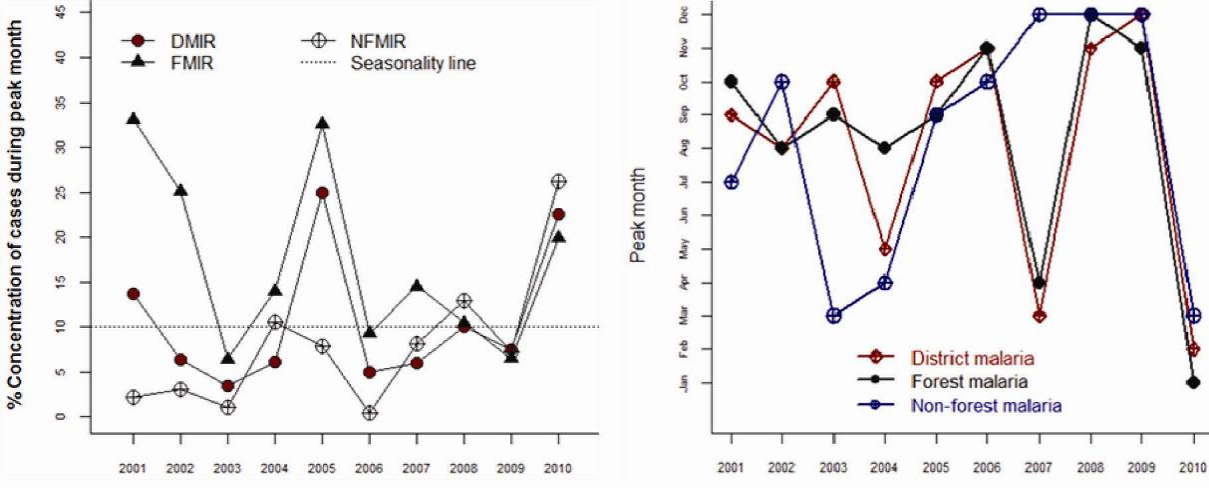
Malaria concentration and peak months of the district

Markham seasonality indices exhibited higher seasonality of concentration of malaria in the forest area than non-forest area (Figure 4). There was no notable displacement of seasonality of concentration of climatic variables; however, seasonality of concentration of malaria in all the three areas used to shift in a random way. Forest malaria was mostly concentrated during the months from August to January, while concentration of non-forest malaria used to roam over the months in both pre-monsoon and post-monsoon malaria seasons. Over the whole period, except in the year 2008, malaria in forest and non-forest areas were seen to concentrate in different months.

### 5.5 Linear Relationships

Overall, weak linear relationships between malaria incidence rates of the three areas and climatic variables were observed during the period (Table 2). However, malaria incidence rates of forest area and non-forest area bore significant linear relationships at 5% significance level with relative humidity components and temperature components respectively. On the other hand, a significant linear relationship was found between malaria incidence rate of the whole district and relative humidity components at 10% significance level. Rainfall did not bear any significant linear relationship with all the three malaria incidence rates.

**Table 2:** Significant linear relationships between MIR and climatic variables

Dependent Variable	Independent Variable	R square	Sig.
DMIR	RH (M)	.026	.080*
	RH (Mean)	.026	.081*
	RH (M)	.045	.021**

FMIR	RH (E)	.049	.015**
	RH (Mean)	.052	.012**

NFMIR	Temp (max)	.050	.014**
	Temp (mean)	.034	.043**

* : sign. at 10% level;

** : Sign. at 5% level.

### 5.6 Models for Estimating MIR

The periodogram of rainfall, temperature and relative humidity suggested 12-month seasonal oscillations. Hence, second term of model (4.0) takes the form β sin (0.52 R_t_T_t_H_t_). Correlogram of seasonally adjusted malaria incidence rate of the whole district (DMIR_ SAS) exhibited significant autocorrelations enduring up to lag 7, while partial autocorrelations at the first two lags only were significant (PACF=0.578 & 0.223); suggesting that each value of DMIR was influenced by the first two preceding consecutive values. The auto.arima function of R suggested an ARIMA model (0,1,1) for designing DMIR and accordingly a white noise residual series was obtained from DMIR by employing ARIMA(0,1,1). A residual series was obtained from sin(0.52RTH) series by employing the same arima model to it and cross correlation test between the two residual series showed that the climatic series was significantly correlated with DMIR lagging one month and five months (coefficients were -0.235 and 0.202). Taking the convenience issue of projection into account, significance at lag (-5) was ignored and significance at lag (-1) only was considered for modeling. Consequently, together with the term β sin(0.52 R_t_T_t_H_t_) representing the influence of climatic variables on the incidence rate, adding two autoregressive terms and a constant term, the model (4.0) takes the form-


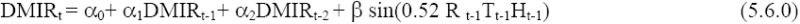


Adjusting the coefficients of the predictor terms of (5.6.0) by weighted least squares regression over the variable values up to the year 2009, the final model for DMIR came out to be-


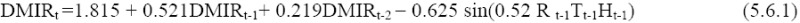


where DMIR_j_ was seasonally adjusted monthly malaria incidence rate of the whole district at time j, and R _t-1_, T_t-1_, H_t-1_ were observed values of rainfall, temperature and humidity respectively at time t*-*1.

For FMIR, instead of seasonally adjusted rate (FMIR_SAS), the square root of FMIR_SAS was found to exhibit significant correlation with climatic variable series at lag (-1). Following the same procedure as in 5.6.1, the model for estimating FMIR came out as-


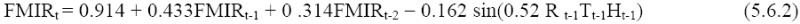


where FMIR_t_ stands for square root of FMIR_SAS at time *t* and other terms carried the same notions as in 5.6.1.

However, for NFMIR, the square root of NFMIR _SAS was found to exhibit a significant correlation with the environmental variable only if the product of seasonally adjusted rainfall and rainy days (both additive model) was considered instead of rainfall. The model for estimating NFMIR, following the same procedure of preceding models, was obtained as-


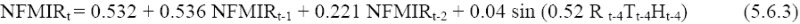


where NFMIR_t_ stands for the square root of NFMIR_SAS, T is the seasonally adjusted mean temperature and H is the seasonally adjusted mean relative humidity. However, in this case, R represents the product series of seasonally adjusted rainfall and rainy days (both additive model).

## 6. Discussion

### 6.1 Role of Climatic Variables

The lowest mean monthly value (LV) and highest mean monthly value (HV) of climatic variables and MIRs, along with their occurring months, over the period 2001-2010 were as in Table 5.

**Table 3:** Observed and estimated malaria rates for the year 2010

	Whole district (DMIR)		Forest area (FMIR)		Non-forest area (FMIR)	
Month	Observed rate	Estimated rate	Observed rate	Estimated rate	Observed rate	Estimated rate
January	6.197	6.301	3.894	3.41	1.811	1.897
February	6.734	6.203	3.413	3.632	2.217	1.913
March	6.086	6.682	3.177	3.597	2.300	2.125
April	5.150	6.826	3.101	3.436	1.853	2.226
May	3.299	6.434	2.028	3.388	1.740	1.995
June	2.740	4.761	2.045	2.761	1.495	1.869
July	2.126	4.527	2.001	2.572	1.201	1.73
August	2.153	3.482	2.310	2.421	1.098	1.532
September	2.604	2.818	2.296	2.428	1.287	1.408
October	3.294	3.096	2.510	2.516	1.528	1.433
November	3.384	4.047	2.815	2.722	1.417	1.631
December	2.204	4.300	2.080	2.935	1.273	1.617

**Table 4:** Model statistics

Dependent variable	Model term	Regression Coefficients	Std. Error	p-value	95% CI
DMIR	Constant	1.815	0.498	<.0001	0.828–2.803
	DMIRt-1	0.521	0.096	<.0001	0.330–0.712
	DMIRt-2	0.219	0.087	0.013	0.047–0.391
	sine(Tt-1R t-1Ht-1)	-0.625	0.210	0.004	-1.042 – -0.209

FMIR	Constant	0.914	0.244	<.001	0.430–1.398
	FMIRt-1	0.433	0.090	<.001	0.255–0.612
	FMIRt-2	0.314	0.086	<.001	0.144 – 0.484
	sine(Tt-1R t-1Ht-1)	-0.162	0.079	.043	-0.318 – -0.005

NFMIR	Constant	0.532	0.173	0.003	0.189–0.874
	NFMIRt-1	0.536	0.096	<.001	0.346–0.727
	NFMIRt-2	0.221	0.094	0.021	0.035–0.407
	sine(Tt-4R t-4Ht-4)	0.040	0.031	0.202	-0.022 – 0.101

**Table 5:** Monthly mean highest and lowest values of variables

Variable	LV	HV	Month of LV	Month of HV
T (mean) (C)	16.78	29.18	January	August
RF (mm)	4.32	792.04	December	July
RD (day)	1	24	December	July
RH (mean) (%)	65.06	86.94	March	July
DMIR (%)	4.59	8.28	February	December
FMIR (%)	8.16	15.97	February	December
NFMIR (%)	3.47	6.43	February	December

The favorable temperature range for development of P. falciparum and P. vivax parasite in India, as mentioned by Bhattacharya et al. (2006), lies between 15°C–35°C (Bhattacharya et al., 2006) (Table 6). Therefore, the temperature of the district remains favorable for transmission of malaria in the district throughout the year. If other conditions do not offset it, malaria parasite may develop every moment in the district.

**Table 6:** Favorable temperature range for malaria parasite development and transmission

Parasites	Class	Transmission window (°C)	No. of days for thriving
*P. vivax*	Class I	15–20	20 ± 5 days
	Class II	20–25	15 ± 5 days
	Class III	25–30	8 ± 2 days

*P. falciparum*	Class I	20–25	25 ± 5 days
	Class II	25–30	20 ± 5 days
	Class III	30–35	10 ± 2 days

Furthermore, the role of rainfall in malaria transmission had been a complex one (Woube, 1997); different studies revealed that rainfall might play both positive (Mendis et al., 1990) as well as negative (De et al., 1990) role in the development of malaria parasites. According to WHO report, moderate rainfall, instead of high volume, was found to be more congenial for malaria incidence (WHO, 1998). Moreover, for Indian malaria, it was observed that rainfall did not directly correlate with malaria (Bhattacharya et al., 2006).

Again, according to Bhattacharya et al. (ibid.), when the average monthly relative humidity remains outside the range of 55–80 percent in India, the life span of malaria mosquito becomes short enough to diminish malaria transmission. From this point of view, the month of January and the period from June to October should not be conducive for the development of malaria mosquitoes in the district as during these months relative humidity remains above 80 percent (81−87 pc). However, malaria incidence persists in the district in moderate degree from June to October. During this period, the mean temperature remains within the optimal range (20°C-30°C) for the development of malaria vector. As relative humidity remains high in the long period of five months, malaria incidence continues to occur as malaria transmitting mosquitoes can live about one month. All this leads to a conclusion that the favorable humidity condition for the development of malaria mosquitoes in the district is a little bit different from the all India average. Malaria mosquitoes can continue to develop in a humidity condition even higher than 80 percent in the district.

In the month of January, prior to the lowest malaria incidence month February, temperature and relative humidity conditions still remain favorable to mosquito growth. However, the mosquitoes do not find available breeding sites such as pool, river, stream, rivulets, spring etc. from November onwards as most of them dry up due to the absent of rainfalls. As a result, malaria incidence remains low during the months of February and March. Rainfall starts towards the last part of February and keeps on increasing; then malaria incidence begins to increase correspondingly. Thus, malaria incidences in both the forest and non-forest areas were significantly correlated with rainfall in the first season of malaria. Initially, due to its structure, the soil surface of forests require more water to fill up its pits, streams, rivulets etc. and usually the land surface of forest area requires more rainwater to get wet than the non-forest surface needs. For this reason, breeding sites are created sooner in non-forest area. So, the impact of high rainfall on malaria incidence is seen sooner in non-forest area. However, excessive rain counteracts mosquito development by flushing out their larvae (Michael & Martens, 1995), and as such malaria incidence in non-forest area comes down by the month of May. However, in the forest area, the growth of malaria mosquito runs steadily, and malaria incidence keeps on increasing till the month of June due to the delayed effect of rainfall in forest area. During the monsoon period, all conditions remain favorable for mosquito development, but rain remains excessive; this keeps malaria incidence in an intermediate range during this period.

During the last malaria season of the year, rainfall almost ceases to occur, but breeding sites still remain available from the rainwater of the previous season. In this season, mosquito development is not obstructed by flushing out of larvae. Moreover, temperature and humidity conditions for the mosquito development remain favorable in this period also. Consequently, malaria incidence stiffly increases in the last malaria seasons in both forest and non-forest areas with the yearly highest incidence occurring in December.

The finding of the current study complies with that of Baruah et al. (2007), which found higher prevalence of malaria during the post-monsoon season in Sonitpur district of Assam, a district from the same state of Assam.

Thus, consideration of all malaria situations and climatic conditions leads to a conclusion that rainfall plays a primary role in characterizing malaria incidences in the district but remains un-reflected in malaria related data.

### 6.2 Model Validation

The model (5.6.1) could explain 45.4% of the observed variability in the malaria incidence rate (R^2^_adj_= 45.4%, F= 30.054, df=3, p<0.001). Histogram of residuals suggested that these were normally distributed with mean zero and standard deviation 1.436. Histogram of the difference between the observed and predicted values of the monthly malaria incidence rate showed that the differences closely followed normal probability distribution with a mean of -0.12 and standard deviation 1.446, which was endorsed by Kolmogorov-Sminov test (p=0.644) also. The scatterplot of the difference between the observed and predicted rates (Figure 5a) shows that only about 1.7% (2/118) of the differences lie above the 95% confidence interval of ±3 cases per 100 examination, and the same number of differences lie below. In addition, the correlation between the difference and observed rate was 0.71 (p<0.001). When the differences were plotted against the observed rates it was found that the slope of the trend line was as small as 0.01. Considering all these results, the model may be considered adequate to forecast the monthly malaria incidence rate of the district in terms of seasonally adjusted rates, from which actual estimated rate may be obtained by multiplying the estimated rate by respective seasonal adjustment factor of the concerning month.

**Figure 5a:**
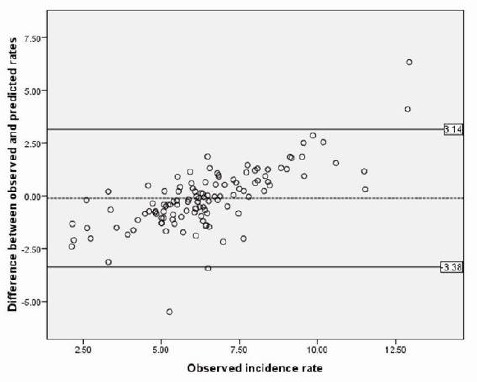
Scatterplot of the difference between observed and predicted rates of DMIR model

The model (5.6.2) for FMIR could explain more than fifty percent of the observed variability in the malaria incidence (R^2^_adj_=50.6%, F=36.785, df=3, p<0.001). Histogram of residuals suggested that these were normally distributed with a mean of -0.003 and standard deviation 0.529. The difference between the observed and predicted values followed a normal probability distribution with a mean of -0.04 and standard deviation 0.553. Kolmogorov-Sminov test also indicated that the distribution did not differ from a normal distribution (p=0.852). The scatterplot of the difference between the observed and predicted rates (Figure 5b) showed that below 1% of the differences (1/118) lie above the 95% confidence interval of ±2 cases per 100 examination, while no difference lie below. The difference and observed rate bore a significant correlation of 0.715 (p<0.001) and the slope of the trend of the difference with respect to the observed rate was 0.002.

**Figure 5b:**
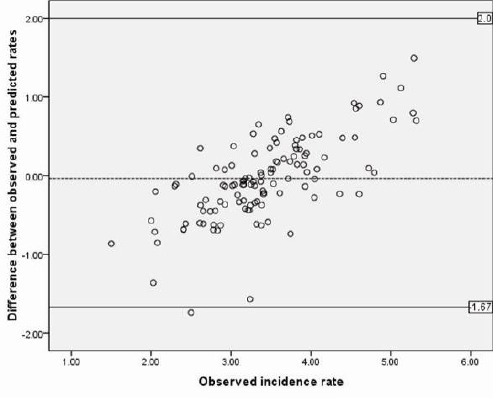
Scatterplot of the difference between observed and predicted rates of FMIR model

The model (5.6.3) for NFMIR could explain 47.2% of the variability in the malaria incidence of the non-forest area (R^2^_adj_=47.2%, F=31.741, df=3, p<0.001). The residuals followed a normal distribution with mean zero and standard deviation of 0.223. The difference between the observed and predicted values of the malaria incidence rate, as observed from their histogram, highly followed a normal probability distribution with a mean of 0.02 and standard deviation 0.232. With a significance of 0.871, Kolmogorov-Sminov test also strongly suggested that the distribution did not differ from a normal distribution. All differences were lying within the 95% confidence interval of ±1 cases per 100 examination (Figure 5c). Correlation between the difference and observed rate was significant (coefficient =0.718, p<0.001), while the slope of the trend of the difference with respect to the observed rate was as small as -0.002. All these results suggested that the model might provide a good estimation of future rate of the monthly malaria incidence of the non-forest area.

**Figure 5c:**
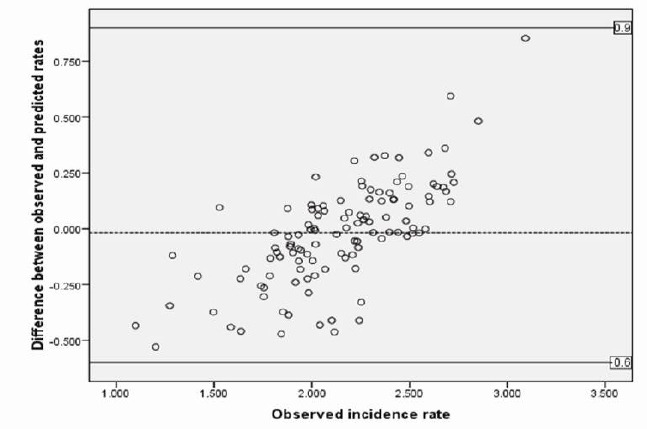
Scatterplot of the difference between observed and predicted rates of NFMIR model

The observed and estimated rates for the year 2010 were as in Table 3, while Table 4 shows the model statistics. Figures 6a, 6b, 6c depict the observed, predicted and estimated rates relating to three models.

**Figure 6a:**
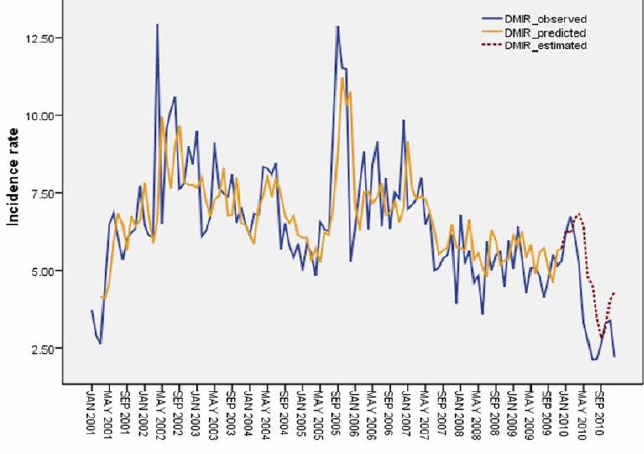
Observed, predicted and estimated incidence rates of DMIR

**Figure 6b:**
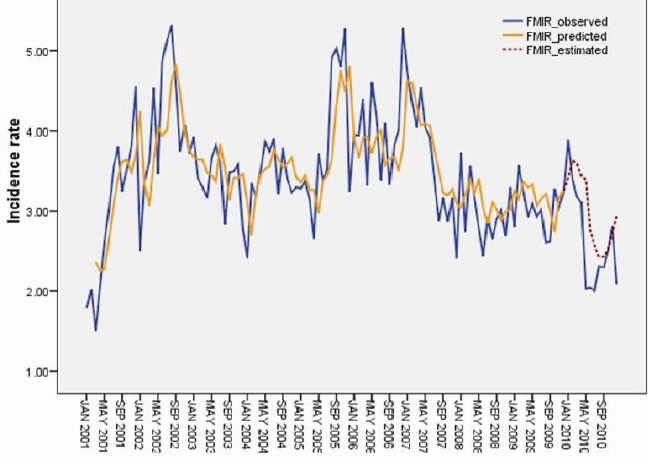
Observed, predicted and estimated incidence rates of FMIR

**Figure 6c:**
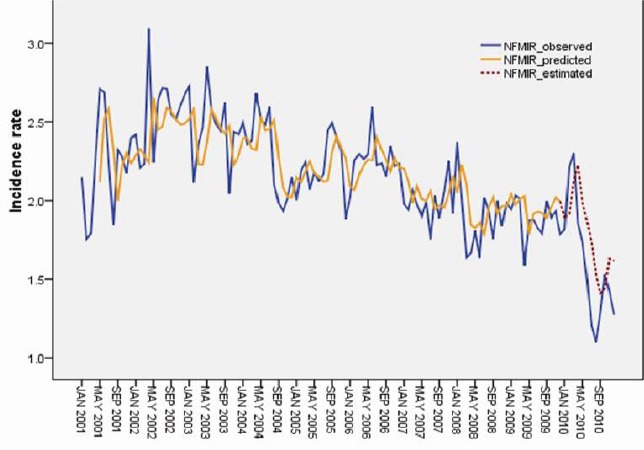
Observed, predicted and estimated incidence rates of NFMIR

The regressions were not performed through the origin, because, from trial and error, it was found that constants were statistically significant in relation to the study data. Earlier, investigation on construction of models for estimating malaria incidence rates of the three areas revealed that instead of actual observed rates, the seasonally adjusted rates could provide more pertinent and reliable model. Series of actual rates contained stiff fluctuations and that resulted in yielding model residuals of great magnitudes. Models with seasonally adjusted rates and their transformations were found to minimize the magnitudes of these residuals to a substantial extent. The only slight inconvenience of the models constructed by these series was that respective retransformations were to be employed to the predicted rates to obtain the actual estimated rates.

### 6.3 Role of Rural Health Program in Controlling Malaria

The Government of India is making effort to carry out architectural correction in the basic health care delivery system of the rural population of the country through a health program called National Rural Health Mission (NRHM). This program has brought a remarkable change in the malaria treatment route of the rural people. Accredited Social Health Activist (ASHA) of NRHM, a trained female community health activist selected from the village itself, is doing a remarkable job in the district in regard to health care of the villagers and treatment of malaria. She collects blood slides from suspected patients for examination of the presence of malaria parasite; these are sent to clinics through a Multi Purpose Worker (MPW), a grass root health functionary for the control of communicable diseases. Then necessary follow up measures are taken for the treatment of malaria if the slide is found parasite positive. Malaria awareness campaigns are organized among the rural people and insecticide treated bed nets are provided for prevention of malaria. There has been a notable decline in malaria incidence in the district after NRHM became fully functional in 2007.

## 7. Conclusion

Climatic variables influence malaria incidence in a complex way in the district. In the beginning of malaria season, they instantaneously facilitate favorable condition for malaria development, and then they normalize the transmission during the middle period, and lastly accelerate it again. Climatic variables are not instantaneous facilitator of malaria transmission in the district. The implicit association between the two makes it difficult to develop a tool for forecasting malaria incidence in the district based on individual influences of the climatic variables and therefore, their combined influence is to be utilized for the purpose. Since climatic variables have different influences on malaria incidence in the forest and non-forest areas, separate measures are required to be adopted for controlling malaria in the two areas. The three models may be expected to yield reliable results in estimating the future malaria incidence rates.

However, it should be admitted that the current analysis has a limitation from the two viewpoints, first, its short data length and second, absence of non-climatic variables in the models. Association between malaria incidence and climatic variables during last ten years only is seemed inadequate to ascertain their future association. Furthermore, malaria incidence is associated with socio-economic conditions of the people, physical condition(s) of the region in concern, and adopted malaria control measures. These factors could not be accommodated in the models.
